# GLS as a diagnostic biomarker in breast cancer: in-silico, in-situ, and in-vitro insights

**DOI:** 10.3389/fonc.2023.1220038

**Published:** 2023-08-18

**Authors:** Danfeng Zhang, Man Wang, Xufeng Huang, Longbin Wang, Ying Liu, Shujing Zhou, Yidan Tang, Qi Wang, Zhengrui Li, Geng Wang

**Affiliations:** ^1^ Departments of Breast Thyroid Vascular Surgery, Taihe Hospital, Hubei University of Medicine, Shiyan, Hubei, China; ^2^ Departments of Outpatient Department, Taihe Hospital, Hubei University of Medicine, Shiyan, Hubei, China; ^3^ Faculty of Dentistry, University of Debrecen, Debrecen, Hungary; ^4^ Faculty of Life Science, Huazhong Agricultural University, Wuhan, China; ^5^ Department of Cardiology, Sixth Medical Center, PLA General Hospital, Beijing, China; ^6^ Faculty of Medicine, University of Debrecen, Debrecen, Hungary; ^7^ Faculty of Medicine, Jiangsu University, Zhenjiang, China; ^8^ Faculty of Dentistry, Shanghai Jiaotong University, Shanghai, China

**Keywords:** breast cancer, bioinformatics, cuproptosis, biomarker, EMT pathway

## Abstract

**Background:**

Recently, a novel programmed cell death mechanism, Cuproptosis, has been discovered and found to play an important role in the development and progression of diverse tumors. In the present study, we comprehensively investigated the core gene of this mechanism, GLS, in breast cancer.

**Materials and methods:**

Bulk RNA sequencing data were curated from the TCGA repository to investigate the aberrant expression of GLS over diverse cancer types. Then, we examined its efficacy as a diagnostic biomarker in breast cancer by Area Under Curve (AUC) of the Receiver Operative Characteristic (ROC) curve. Furthermore, by applying siRNA technique, we knocked down the GLS expression level in cancerous cell lines, measuring the corresponding effects on cell proliferation and metastasis. Afterward, we explored the potential implications of GLS expression in the tumor immune microenvironment quantitatively by using several R packages and algorithms, including ESTIMATE, CIBERSORT, etc.

**Results:**

Pan-cancer analysis suggested that GLS was aberrantly over-expressed in many cancer types, with breast cancer being typical. More in-depth analyses revealed the expression of GLS exerted a high ROC-AUC value in breast cancer diagnosis. Through the knock-down of GLS expression, it was found that GLS expression was strongly relevant to the growth and metastasis of tumor. Furthermore, it was also found to be correlated with the immune tumor microenvironment.

**Conclusion:**

We highlighted that GLS expression might be applicable as a diagnostic biomarker in breast cancer and possess significant implications in the growth and metastasis of tumor and the immune tumor microenvironment, sharing new insights into ontological and personalized medicine.

## Introduction

**Figure 1:**
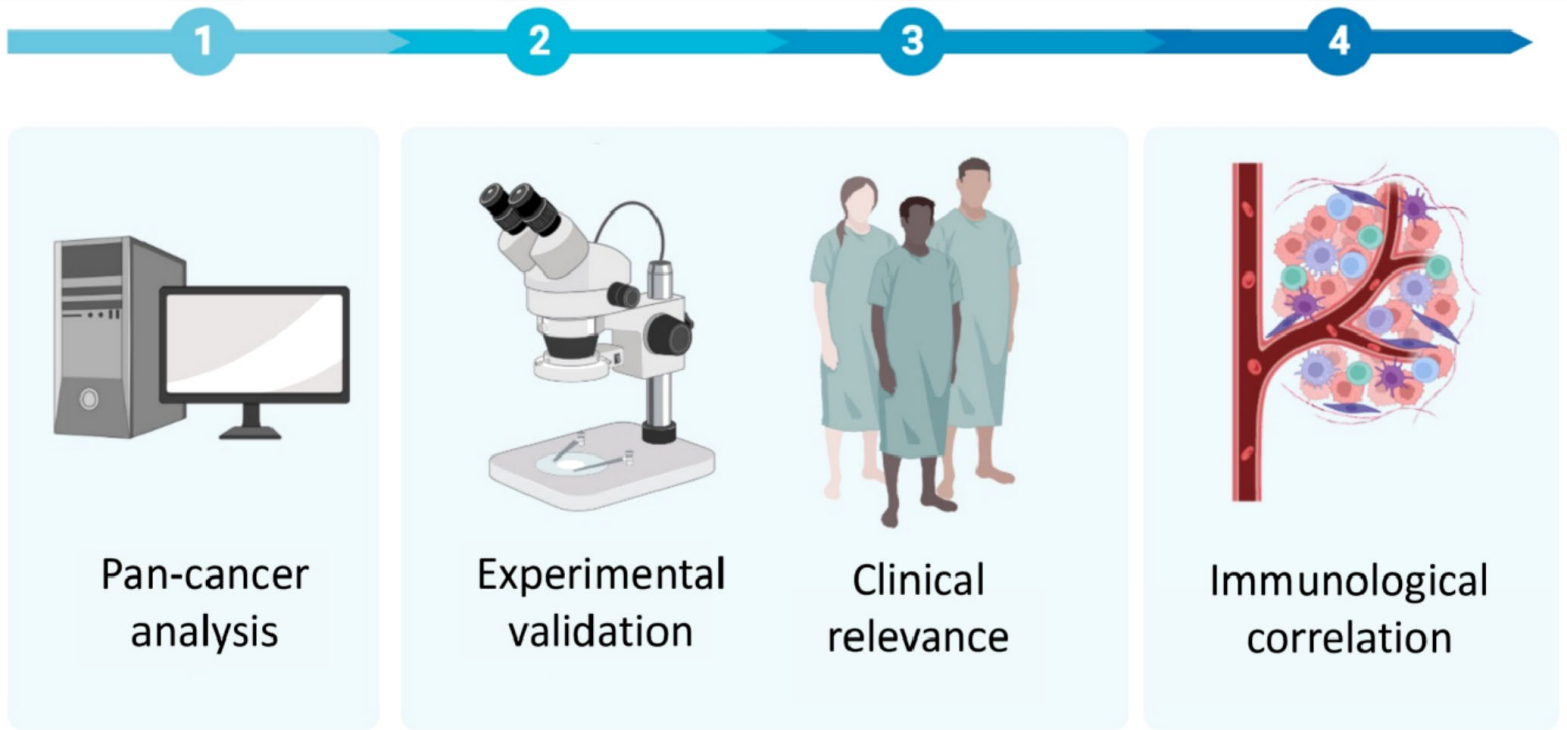
Graphical abstract of the present study.

Breast cancer is the most common malignant tumor in women and one of the main causes of disease burden worldwide (1). Despite the significant advances in diagnosis and prognosis, treating patients with advanced stages remains challenging (2–5). To improve the prognosis, early detection, and timely treatment are of great significance. Therefore, revealing new and highly sensitive molecular biomarkers remains critical. Cuproptosis, a newly discovered cell death mechanism, serves an important role in the modification of specific mitochondrial metabolic enzymes, through which it triggers a series of intracellular signaling events and ultimately leads to cellular caseation (6). Earlier studies have also shown that the aberration of copper levels in the serum and tissues of patients with different solid tumors indicates poor clinical outcomes in a fair number of cases (7–10). Indeed, copper as a basic metal ion in most aerobic organisms serves as a structural and catalytic cofactor for many essential biological enzymes (11). Furthermore, in some studies, certain copper chelators were applied in anticancer therapies, such as tetrathiomolybylate, which has shown a clear survival benefit for high-risk triple-negative breast cancer (12, 13). Therefore, it is of ration that utilizing Cuproptosis-related genes to assist in the early screening of breast cancer. GLS, one of the 10 Cuproptosis-related genes, is an encoding gene of glutaminase which is the enzyme catalyzing the hydrolysis of L-β-glutamine into L-glutamate and ammonia. As glutamine metabolism plays an important role in various tumors, GLS has also become a promising therapeutic target in cancer treatment (14, 15). However, back to date, the utilization of GLS in breast cancer diagnosis remains in need. From this end, it is of interest to explore the role of GLS from this aspect. In the present study, by integrating bioinformatics analytics and immunohistochemical (IHC) staining of real-world patient samples from the HPA database, as well as the siRNA technique, we proposed the idea that GLS may serve as a valuable diagnostic biomarker for breast cancer. **Figure 1** demonstrates the general design of the present study.

## Materials and methods

### Data curation

The sequencing data of 10,534 TCGA pan-cancer samples and 15,776 GTEx normal samples were downloaded from the UCSC Xena database. In particular, for breast cancer, raw transcriptomic count data and the normalized mRNA profile fragments per kilobase of exon per million reads (FPKM) of 115 TNBC patients and 113 normal tissues were retrieved.

### Logistics modeling and diagnostic nomogram construction

The logistics algorithm was used to model the data so that the predictive ability of GLS expression for breast cancer could be further improved. Subsequently, we established a nomogram for predicting the diagnostic probability of breast cancer using the expression of GLS.

### Estimation of the tumor microenvironment condition

The tumor microenvironment condition was assessed quantitatively by calculating the levels of stromal and immune cell infiltration using the expression profiles obtained from the TCGA dataset. This was done by the R package “ESTIMATE” in which the stromal score, immune score, and ESTIMATE score were calculated (16).

### Screening of immune cell infiltration

For the analysis of immune cell infiltration, we used the CIBERSORT algorithm which is a strong and robust immunoinformatic method to evaluate the immune cell population based on bulk RNA transcriptome. This was achieved by the R package “immunedeconv” (17).

### Single-cell analysis

The single-cell transcriptomic profile of the GSE114727 dataset (18) was recorded in the Gene Expression Omnibus (GEO) database. The visualization was done by the online toolkit TISCH (http://tisch.comp-genomics.org/) (19).

### Cell culturing and transfection

The breast cancer cell line, MCF7, was cultured in Huazhong University (April 2023). The medium was specially designed (i.e., DMEM + 10% FBS + 20ng/ml EGF + 0.5ug/mL Hydrocortisone + 10ug/mL Insulin + 1% NEAA + 1% P/S) and regularly changed when the medium colour turned yellowish. The culturing flasks were saved consistently at 37°C in a humidified incubator containing 5% CO2.

The vectors, si-NC and si-GLS were transfected into MCF7 cell line by Liposome 2000 transfection agent (Invitgen, USA) according to the manufacturer’s protocol. Sequences of si-NC was: UUCUCCGAACGUGUCACGUTT ACGUGACACGUUCGGAGAATT, and sequences of si-GLS was: GAUGGUGUCAUGCUAGACAAATT UUUGUCUAGCAUGACACCAUCTT.

### CCK8 assay

The siRNA-transfected MCF7 cells were fully digested, inoculated into 96-well culture plates at 2 x 10^3^ cells per well and incubated at 37°C. The absorptivity was measured every day using the CCK-8 kit, for a total of seven days of incubation. The above procedures were carried out at 450 nm on the advice of the reagent supplier and four secondary wells were retained for the experimental sessions.

### Western blotting

We treated the MCF7 cell line with cell lysis buffer at 100°C for a total of 10 min with cells that had been previously rinsed with cold PBS to extract total protein. Prepared proteins were added to a 10% SDS/PAGE grid system and equal amounts of proteins were electrolysed at constant pressure (i.e., 200V) and then transferred to a PVDF membrane. After sealing the membrane with 5% skimmed milk powder for 1h, we incubated it overnight at 4°C with primary antibodies, followed by 1.5h incubation with secondary antibodies. Then, the PVDF membrane strips were washed and visualized, the relative protein abundance was assessed using Image Lab analysis software and ImageJ.

### Statistical analysis

R Studio software (v 4.1.0) was used for bioinformatics analytics and GraphPad Prism 5.0 (GraphPad Software, Inc., San Diego, CA) was used for semi-quantification of the real-time PCR and western blot results. The software was also used to generate the relevant charts. The Chi-square test or the Wilcoxon signed-rank test was conducted to calculate the P-values. The correlation analyses were done under the Spearman method. The area under the curve (AUC) and the receiver operating characteristic (ROC) curve were analyzed to determine the predictive accuracy. P-value < 0.05 was deemed statistically significant.

## Results

### Pan-cancer analysis of GLS expression across various cancer types

**Figure 2:**
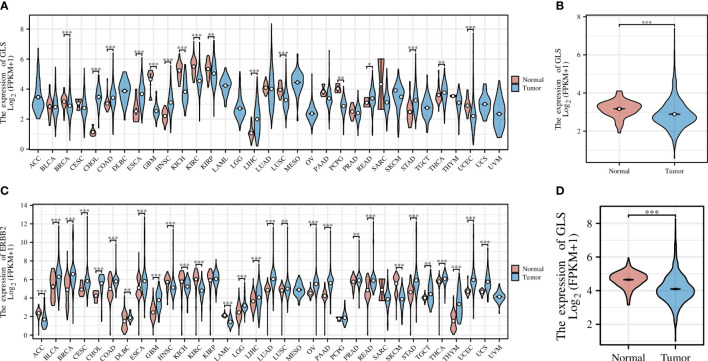
Pan-cancer analysis of GLS expression across various cancer types, with the **(A)** TCGA dataset alone and **(C)** combined it with the GTEx data. **(B, D)** Demonstrate the comparison of GLS expression in normal samples and tumor samples, respectively. *P < 0.05, **P < 0.01, ***P < 0.001.

Pan-cancer analysis is a widely used and efficient approach to identify the diagnostic value of a specific gene in regard of human tumors (20, 21). Therefore, we first conducted a pan-cancer analysis based on the transcriptomic data from the TCGA database, the result of which revealed that the expression of the GLS gene was significantly down-regulated in diverse cancer types, including but not limited to GBM, KICH, KIRC, and KIRP, etc. (**Figure 2A**), particularly in the breast cancer (abbreviation: BRCA or BC, **Figure 2B**). As the TCGA database lacks normal controls, we further combined the transcriptomic data from the GTEx repository for comparison. As a result, the previous observations were confirmed (**Figures 2C, D**).

### Evaluation of the reliability of GLS expression as a diagnostic biomarker in breast cancer

**Figure 3:**
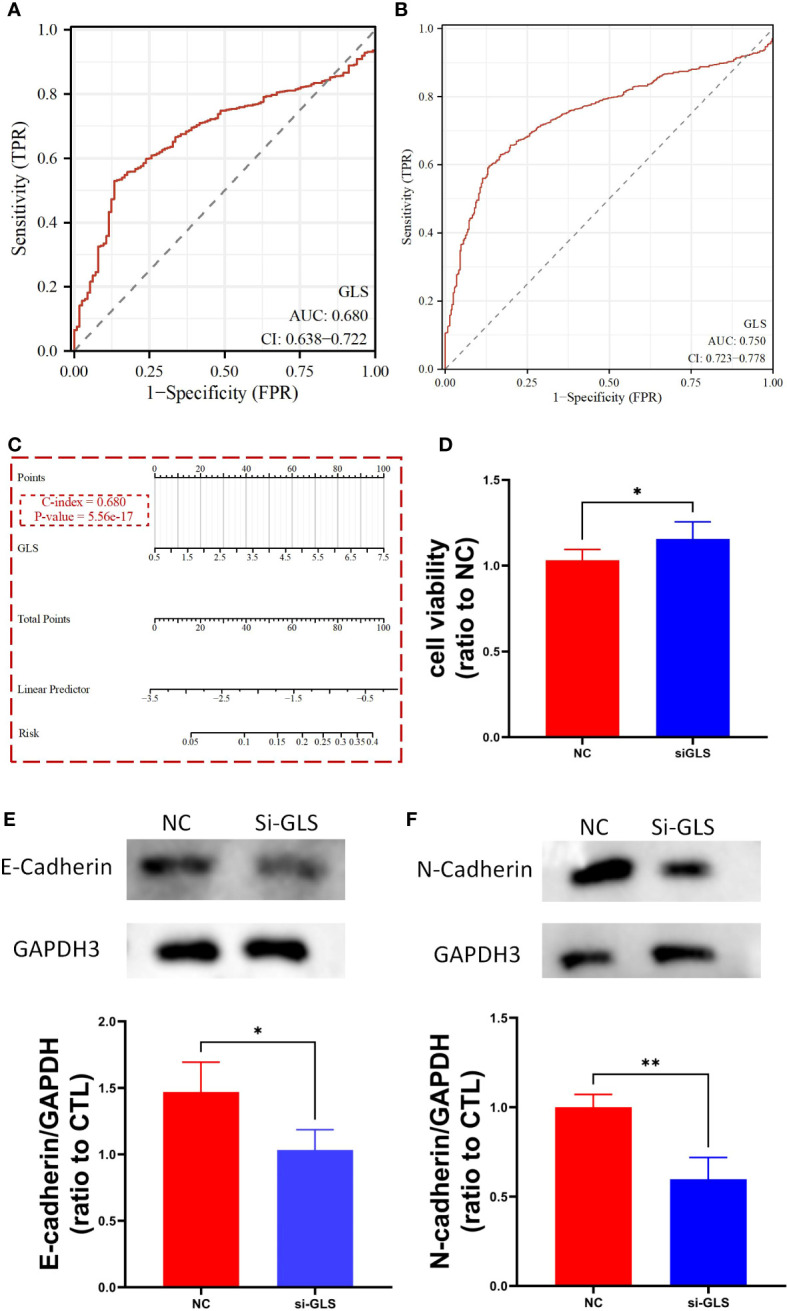
GLS expression might serve as a diagnostic biomarker for breast cancer. **(A, B)** Demonstrate the reliability of using GLS expression as a diagnostic biomarker in breast cancer by ROC curves, respectively. **(C)** Diagnostic nomogram utilizing GLS expression as an indicator for breast cancer. **(D)** Is the cck8 result demonstrating the comparison of controlled group (i.e., NC) and siRNA group (i.e., si-GLS) from the aspect of cell viability. In contrast with the controlled group, when GLS expression was impaired, cell viability was significantly better. **(E, F)** Are the western blot images of the 2 key emt pathway proteins (i.e., E-cadherin and Ecadherin) with semi-quantification, respectively. *P < 0.05, **P < 0.01.

We used the TCGA dataset alone (**Figure 3A**) and combined it with the GTEx database data (**Figure 3B**) to plot the receiver operating curves (ROC curves), through which we found that the ROC-AUC values were high (in the case of solely used TCGA dataset, ROC-AUC value = 0.68, and in the case of TCGA-GTEx combination, ROC-AUC value = 0.75), suggesting that GLS may serve as an important diagnostic biomarker breast cancer. Then, we used the logistics algorithm to optimize a model aiming for better diagnostic prediction and constructed a GLS-based nomogram (**Figure 3C**), with a C-index = 0.680, hindering it possessed a very good clinical diagnostic ability, which once again supported our idea.

By knocking down the GLS expression *via* the siRNA techinique, we found that the down expression of GLS promoted the vitality of tumor cells (**Figure 3D**).

As we know, the emt pathway has a very important role in cancer deterioration. From the very general aspect, the up regulation of emt pathway is regarded as one of the potential bases of tumor metastasis. On the other hand, E-adherin and N-cadherin, the 2 key proteins in the emt pathway are thought as the most active players that anchor the tumor tissues to their place of origin. Namely, when the 2 proteins are down regulated, the emt pathway will be activated, and so more likely to have tumor metastasis occurred. Our observation showed that after intervening with si-GLS, the expression levels of both E-cadherin and N-cadherin were significantly decreased, indicating a promoting effect on the pathway, and so the metastasis of the tumor (**Figures 3E, F**).

### The expression of GLS was tightly associated with multiple clinical characteristics of breast cancer

**Figure 4:**
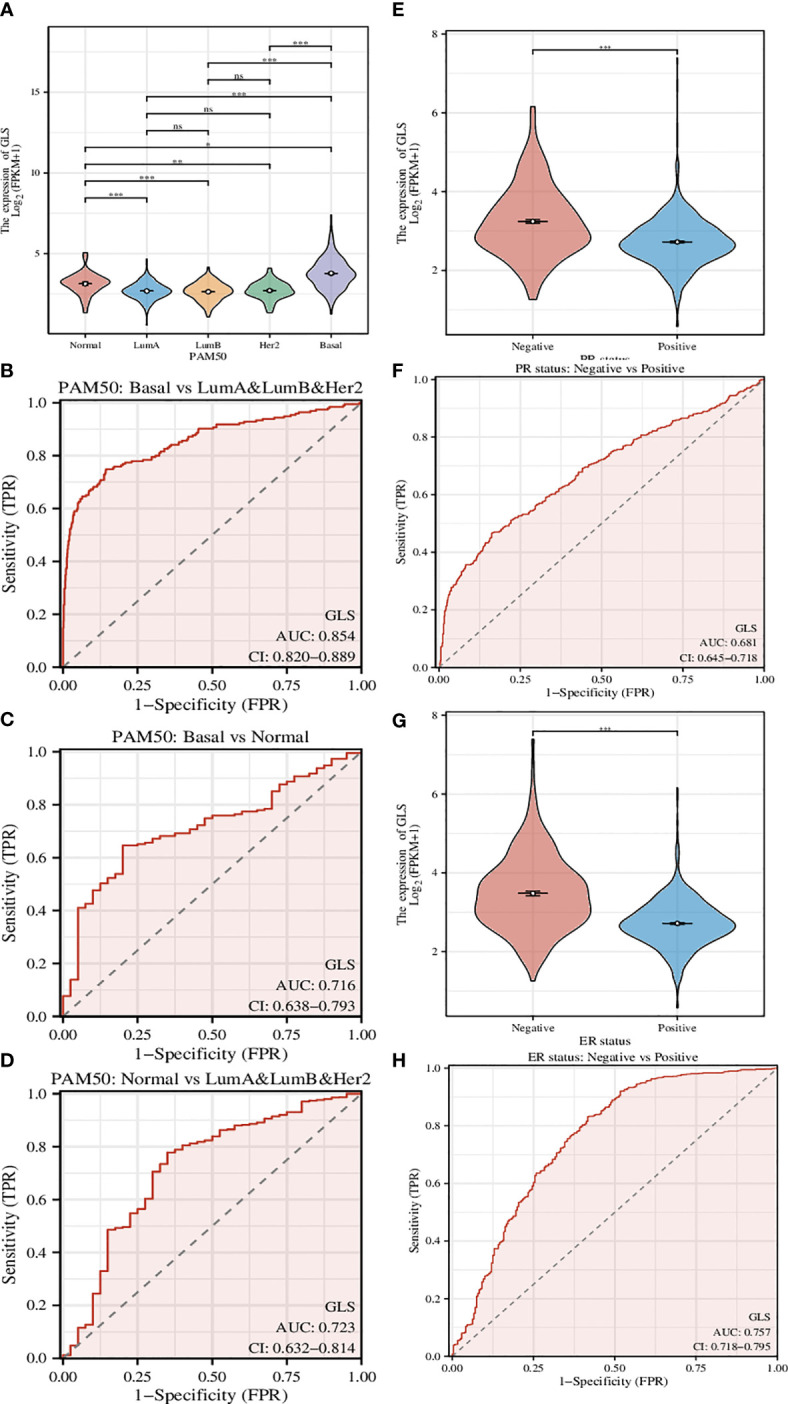
Investigation of the correlation between different clinical characteristics and the GLS expression. **(A)** Utilization of GLS expression to classify the molecular subtypes of breast cancer. **(B-D)** ROC curves to assess the reliability of **(A)**. **(E, F)** Utilization of GLS expression to classify the PR positive and negative subtypes of breast cancer, and the ROC curve to assess the reliability. **(G, H)** Utilization of GLS expression to classify the ER positive and negative subtypes of breast cancer, and the ROC curve to assess the reliability. *P < 0.05, **P < 0.01, ***P < 0.001.

A genetic signature coined as PAM50 composed of 50 genes is commonly used clinically to divide breast cancer into several different molecular subtypes, and even now is still being a critical indicator in the treatment of breast cancer (22). Here we used GLS instead of this signature (**Figure 4A**) and found that the classification can be performed more efficiently, although the classification is somehow rougher (Luminal type A, Luminal type B, and Her2 type were indistinguishable, thus hereby we termed it as mixed type). The ROC analyses of the comparison between Basal type and mixed type (**Figure 4B**), between Basal type and Normal type (**Figure 4C**), and between Normal type and mixed type (**Figure 4D**) were also conducted. As a result, we found that such GLS-based distinguishments were of high accuracy with high ROC-AUC values, especially in the case of Basal type and mixed type, the ROC-AUC value was up to 0.854.

PR-positive cancer cells may require progesterone to grow. Consequently, these cells may stop growing or die when treated with substances that block the binding and action of progesterone. Therefore, patients with positive PR in the short term have better treatment prospects. In this study, we found that GLS expression was significantly reduced in PR-positive patients than in PR-negative patients (**Figure 4E**). This conjecture was then confirmed by the subsequent ROC analysis results (AUC-value = 0.681, **Figure 4F**).

Similar to PR status, ER-positive cancer cells may require estrogen to grow. These cells may stop growing or die when treated with substances that block the binding and action of estrogen. Therefore, patients who are ER-positive in the short term have better treatment prospects. We found that GLS expression was also significantly less in ER-positive patients than in PR-negative patients (**Figure 4G**). This conjecture was also confirmed by the subsequent ROC analysis results (AUC-value = 0.757, **Figure 4H**).

Taking them altogether, based on the PR/ER status, it seems that the low expression of GLS may exert adverse effects on the treatment in the short term. In addition, its expression level in breast cancer is lower than that in normal tissues, as such, finding ways to increase its expression level may be a new adjuvant treatment strategy.

### Exploration of the immune implications of GLS in breast cancer

**Figure 5:**
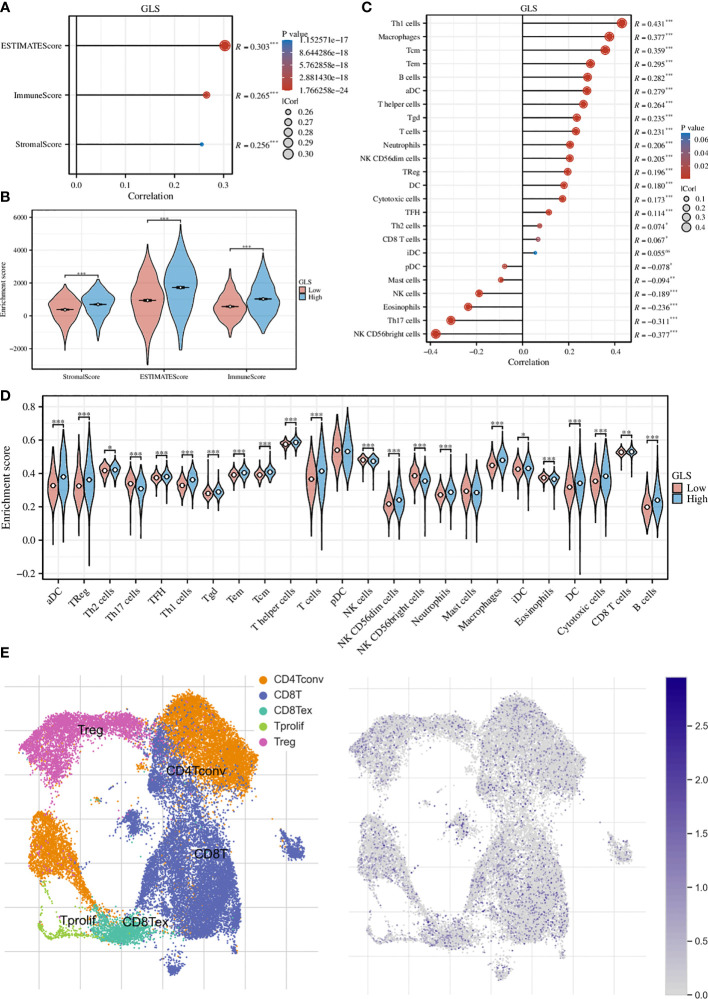
Exploration of the immunological implications. **(A)** Correlation analysis of GLS expression and the ESTIMATE, stromal, and immune scores. **(B)** Comparison of the ESTIMATE, stromal, and immune scores in high and low GLS expression groups. **(C)** Correlation analysis of GLS expression and the infiltration of different immune cell types. **(D)** Comparison of immune cell infiltration in high and low GLS expression groups. **(E)** Visualization of the expression of GLS in various immune cells at the single-cell level. The left panel demonstrated the cell populations in the GSE114727 dataset which described the general tumor immune microenvironment in breast cancer, and the right panel showed the GLS expression of each cell within the dataset. *P < 0.05, **P < 0.01, ***P < 0.001.

We first assessed the association of GLS with the tumor microenvironment by using the ESTIMATE algorithm and found that it was significantly associated with the stromal cells and immune cells (**Figure 5A**). This conclusion was further strengthened by the group comparison between high- and low-GLS expression (**Figure 5B**). Then, we performed ssGSEA analysis on a variety of immune cells and the expression of GLS. Subsequently, it was found that GLS extensively affected the infiltration of almost all immune cell types (**Figure 5C**). This conclusion was also further strengthened by the group comparison of the expression level of GLS (**Figure 5D**), as well as its visualization at single-cell transcriptome (**Figure 5E**), which further highlighted the important impact of GLS on the immune microenvironment of breast cancer.

### Functional analysis

**Figure 6:**
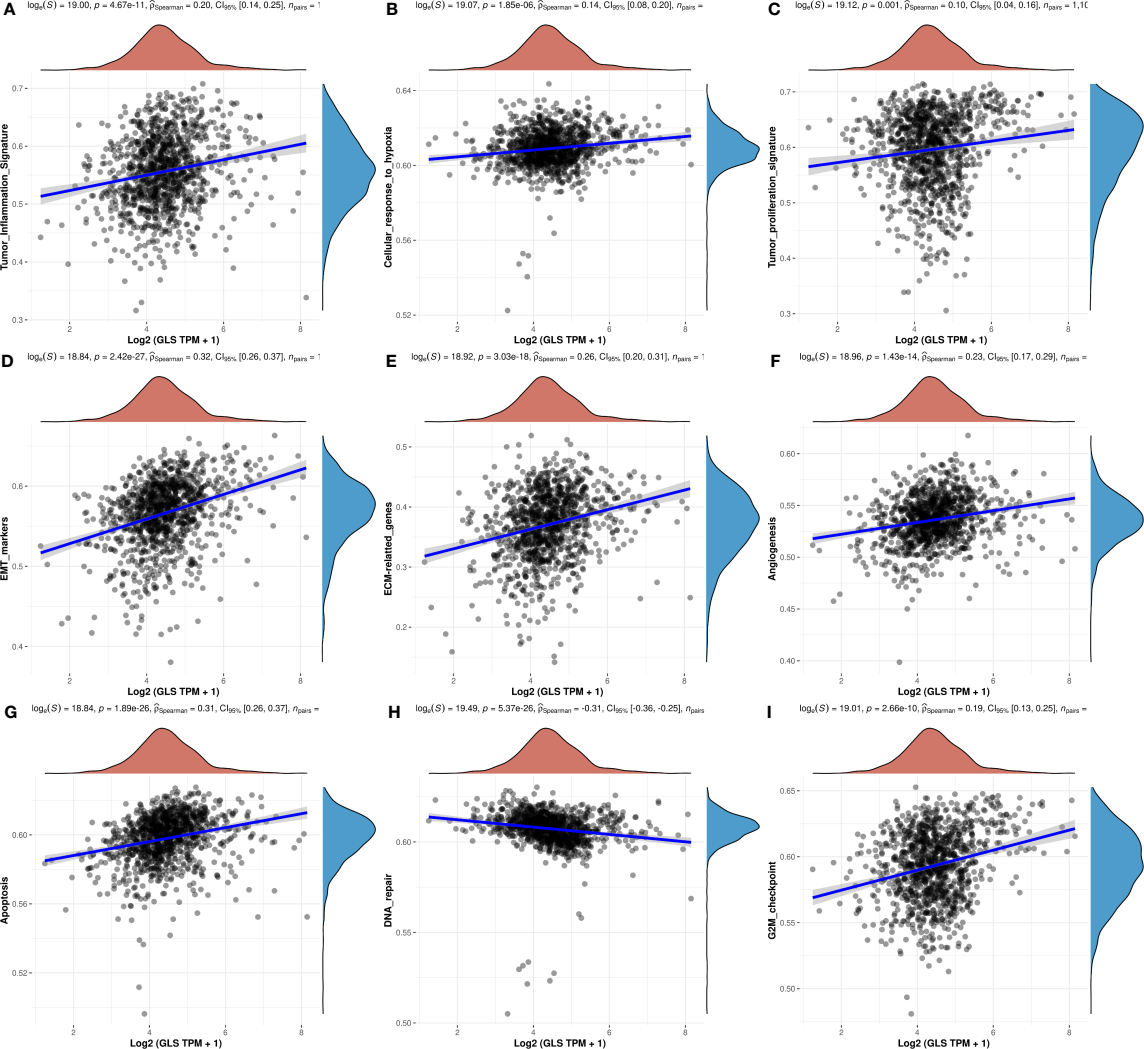
Investigation of the functionalities of GLS in breast cancer. Correlation between GLS expression and Tumor Infiltration Signature **(A)**, Cellular response to hypoxia **(B)**, Tumor proliferation signature **(C)**, EMT markers **(D)**, ECM-related genes **(E)**, Angiogenesis **(F)**, Apoptosis **(G)**, DNA repair **(H)**, G2M checkpoint **(I)**, respectively.

Through the ssGSEA algorithm, we investigated the functionalities of GLS in breast cancer. It was found that except for DNA repair (**Figure 6H**), its expression was positively associated with the rest pathways (**Figures 6A-G, I**).

In addition, we supplemented an analysis of drug sensitivity targeting the GLS gene based on the GDSC repository (**Supplementary Figure S1**), from that we found KIN001−102, Phenformin, WZ3105, Vinorelbine, and SB52334 were positively related to GLS expression. They might have certain value to be tested in the future.

## Discussion

Breast cancer is a life-threatening disease that poses a significant challenge to the world and has been becoming a pivotal cause of global women’s cancer death (1). Although the survival outcomes have significantly improved over the past decades, patients with metastatic breast cancer still show poor prognoses (2–5). As such, early detection and genetic screening of breast cancer are of great interest to public health.

On the other hand, Cuproptosis, a novel cell death mechanism induced by copper ion imbalance, has been shown to play a significant role in the development and deterioration of various cancers, indicating that it could serve as a potential biological target for diagnosing or treating these illnesses (6–13). Meanwhile, GLS, the gene encoding glutaminase, is an important Cuproptosis-related gene. The dependence of a plethora of malignancies on glutaminase has prompted the proposition of glutaminase inhibitors as a viable therapeutic strategy for cancer (14, 15).

To date, although previous studies have shown that imbalanced copper metabolism can significantly impact the tumor microenvironment, adjusting the immunotherapy responsiveness, and several researchers even established GLS-involved signatures associated with human cancer cell proliferation and metastasis, there is still a lack of focus on the correlation between GLS and breast cancer. From this end, the present study explored the possibility of using GLS as a diagnostic biomarker for breast cancer and further clarified the underlying mechanisms by the bioinformatics method combined with clinical investigations on real-world samples.

We first conducted a pan-cancer analysis, reviewing its expressions in different cancers. As a result, its aberrant expressions were observed in diverse cancer types, especially breast cancer. In breast cancer, it was found that GLS was abnormally expressed which raised our interest in the possibility of utilizing it as a diagnostic biomarker in the future. In fact, the ROC-AUC values in the TCGA cohort alone and combination with GTEx data were surprisingly satisfying. Therefore, we further modeled a diagnostic nomogram aiming to assist physicians in making clinical decisions in their daily practice. More important, we surprisingly found that the expression of GLS could be used for a more efficient classification of molecular subtypes in breast cancer, although certain more exact subtypes were not applicable.

Given that nowadays the detailed interplays of the tumor immune microenvironment (TIME) have been unraveled drastically, its major component, namely the vast diversity of immune cell populations such as regulatory T cells, cytotoxic T cells, helper T cells, B cells, monocytes, etc. are deemed to play a critical role regarding various diseases, including cancers (23–26). Therefore, it was of huge interest to investigate the TIME in the present study. To the best of our knowledge, this is the very first time to explore the relationship between GLS expression and the TIME in breast cancer. We first utilized the ESTIMATE algorithm to examine the association of GLS expression with the TIME. Through quantitatively measuring the stromal and immune landscapes by the ssGSEA method, we found that the expression of GLS possessed statistically significant impacts in both regards, which was further supported by the comparison of high and low GLS expression groups. More importantly, at the single-cell level, we found its expression was enriched in various immune cells including CD8+ and CD4+ T cells, B cells, and more. These findings suggested that GLS might extensively involve in the shaping of the tumor microenvironment in breast cancer.

In conclusion, in the present study, we performed in-depth bioinformatics analytics of GLS expression in breast cancer, based on which we established a practically useful diagnostic monogram, which to a large extent, cross-supporting the previous research, although form a different aspect (27). Collectively speaking, the idea of using GLS as a biomarker for breast cancer is convincing. Moreover, we provided significant mechanistic insights into GLS’s impacts on the tumor microenvironment, focusing on the immune aspect.

## Data availability statement

The original contributions presented in the study are included in the article/**Supplementary Material**. Further inquiries can be directed to the corresponding author.

## Author contributions

Conceptualization: XH, DZ, QW, ZL, and GW. Data curation: XH, DZ, MW, SZ, LW, and YT. Formal analysis: XH and DZ. Visualization: XH, YL, SZ, QW and ZL. Writing – original draft: XH, DZ, MW, LW, SZ, YT, QW, ZL, YT, and YL. Writing - review and edition: YL and GW. All authors contributed to the article and approved the submitted version.

## Supplementary material

Supplementary Figure S1 Chemosensitivity test targeting GLS gene expression.
