# Supplementary figures and images for: GLS as a diagnostic biomarker in breast cancer: in-silico, in-situ, and in-vitro insights
Source: Front Oncol. 2023 Aug 18;13:1220038. doi: 10.3389/fonc.2023.1220038 (PMC10471892; doi:10.3389/fonc.2023.1220038)

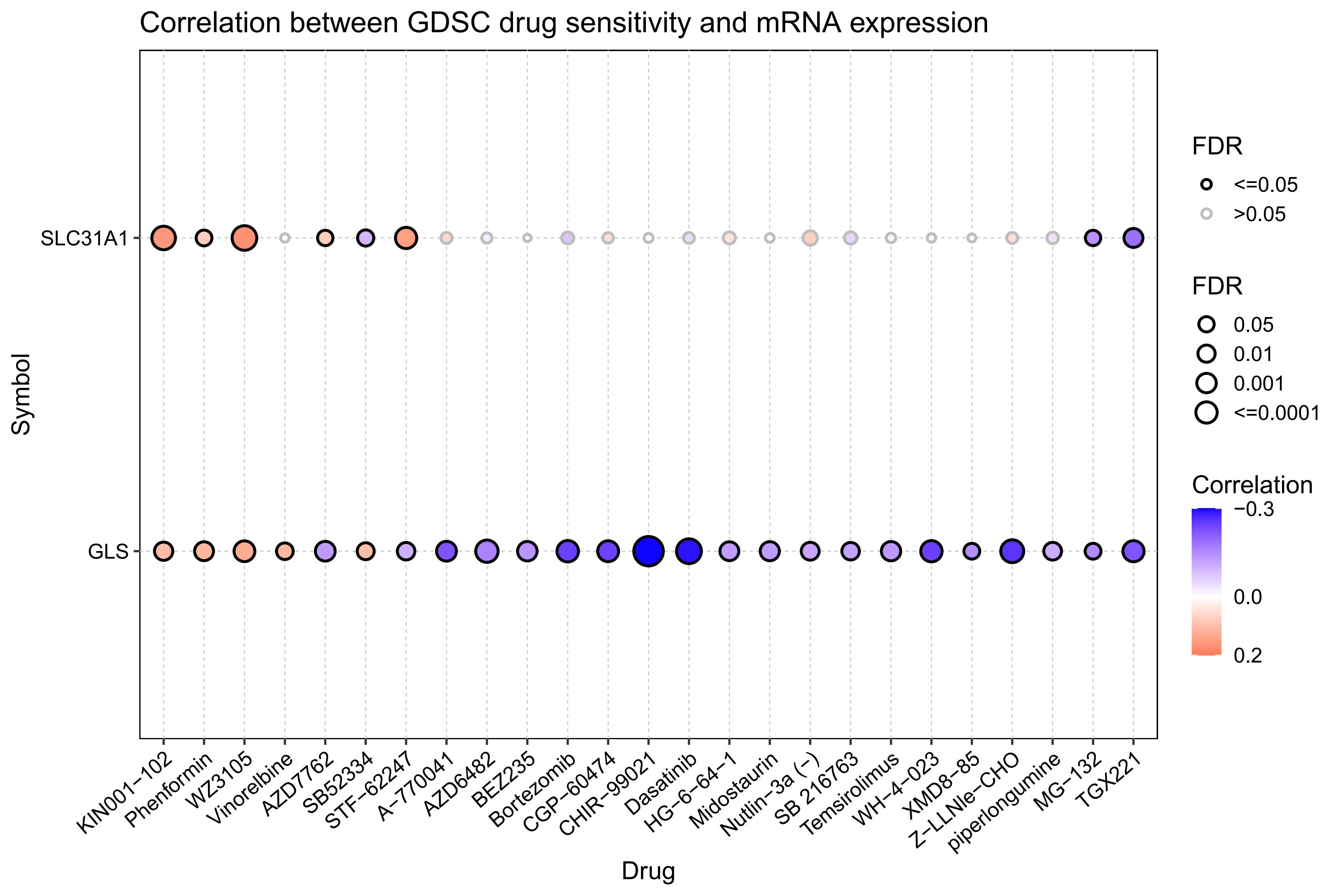

Supplement: Supplementary Figure S1 — Chemosensitivity test targeting GLS gene expression. [file Image_1.jpeg]
